# Supplementary material for: An Evaluation of the Impact of an OPEN Stewardship Generated Feedback Intervention on Antibiotic Prescribing among Primary Care Veterinarians in Canada and Israel
Source: Animals (Basel). 2024 Feb 16;14(4):626. doi: 10.3390/ani14040626 (PMC10885889; doi:10.3390/ani14040626)
Supplement: Supplementary file 1 [file animals-14-00626-s001.zip › animals-2813049-supplementary.pdf]

Supplementary Materials

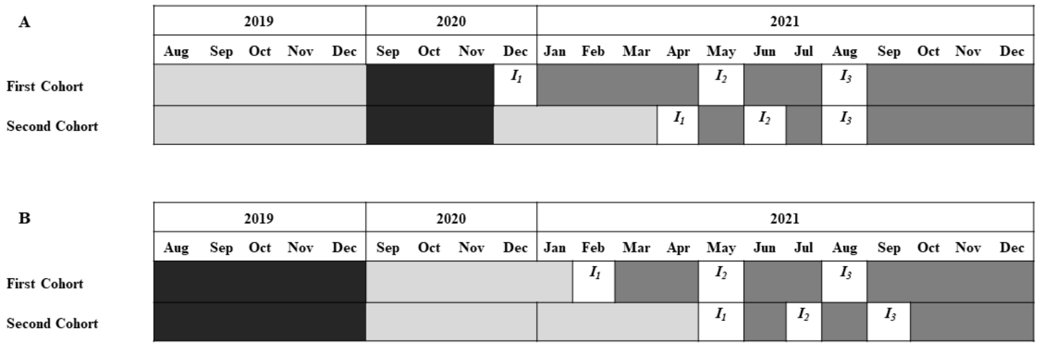

Figure S1. Timeline of the study for the first and second cohort of participants in (A). Israel and (B). Canada. Grey shading represents the months pre-intervention, black shading represents months when no data were collected, no shading represent the intervention months, and dark shading represent the months post-intervention. I1 : First intervention (Critical antibiotics use report), I2: Second intervention (Duration of antibiotics use report), I3 :. Third intervention (Broad Spectrum antibiotics use report).

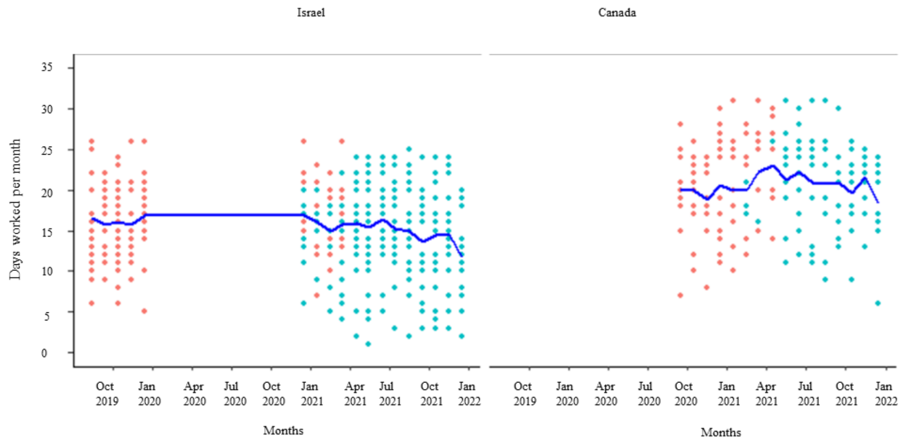

Figure S2. The monthly number of days worked by the participants during the study period before (red dots) and after (blue dots) the receipt of the first feedback report. The blue lines represent the mean number of days worked in each country.

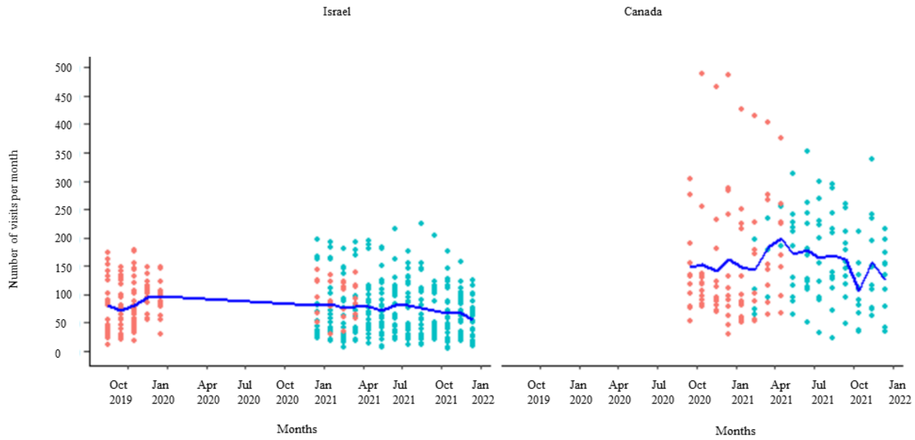

Figure S3. The number visits per month during the study period before (red dots) and after (blue dots) the receipt of the first feedback report. The blue line represents the mean number of visits per month.

Table S1. Summary statistics of the veterinarians' monthly antibiotics prescribing, days worked and total visits during the study period.

Summary statistics	Monthly antibiotics prescribing					Days worked per month	Total visits per month
	Critical	Other	Broad-spectrum	Narrow-spectrum	Total		
Min	0.00	0.00	0.00	0.00	1.00	1.00	5.00
First quartile	6.00	4.00	4.00	5.00	10.00	13.00	46.25
median	11.00	6.00	7.00	10.00	18.00	17.00	86.00
Mean	13.55	9.20	9.11	13.64	22.75	16.90	100.41
Third quartile	18.00	12.00	13.00	17.00	31.00	21.00	129.00
Max	103.00	68.00	78.00	139.00	160.00	31.00	488.00
